# The utilization of microbial cell-free DNA next-generation sequencing for the detection of human herpesvirus-8 in a quaternary care center

**DOI:** 10.1177/20499361251313832

**Published:** 2025-01-23

**Authors:** Rebecca Berger, Jennifer Jacobe, Fernando H. Centeno, Todd Lasco, Mayar Al Mohajer

**Affiliations:** School of Medicine, Baylor College of Medicine, 1 Baylor Plaza, Houston, TX 77030, USA; School of Medicine, Baylor College of Medicine, Houston, TX, USA; Department of Medicine, Baylor College of Medicine, Houston, TX, USA; Department of Pathology, Baylor College of Medicine, Houston, TX, USA; Department of Medicine, Baylor College of Medicine, Houston, TX, USA; Baylor St. Luke’s Medical Center, Houston, TX, USA

**Keywords:** clinical utility, HHV8, immunocompromised, mcfDNA nGS

## Abstract

**Background:**

Human herpesvirus-8 (HHV8) can present with cutaneous or extracutaneous manifestations. While violaceous skin lesions characterize cutaneous Kaposi sarcoma, extracutaneous HHV8 is challenging to diagnose due to nonspecific symptoms.

**Objectives:**

We evaluated the role of microbial cell-free DNA next-generation sequencing (mcfDNA NGS) in diagnosing HHV8-related illness.

**Design:**

Retrospective analysis.

**Methods:**

Between 2017 and 2024, we reviewed the medical charts of 10 immunosuppressed patients at a quaternary care center who had positive HHV8 mcfDNA NGS results.

**Results:**

The clinical and laboratory turnaround times of mcfDNA NGS were 3 and 1 days, respectively (8.5 and 7 days for immunohistochemistry vs 5.5 and 2 days for serum HHV8 polymerase chain reaction). Eight patients received HHV8-related diagnoses, while two had unrelated conditions. Management changed in six patients post-testing due to outpatient specialist referral or adjusting antimicrobials.

**Conclusion:**

mcfDNA NGS can aid clinicians in identifying HHV8-related diseases before tissue sampling and adjusting treatment plans in patients with nonspecific disease manifestations.

## Plain language summary

Next generation sequencing for HHV8 detection

Why was the study done? Doctors often have difficulty figuring out what infection an immunocompromised person has. This is because the symptoms can be vague, and there are many possible culprits. Human Herpesvirus 8 often affects people with a weakened immune system and can cause a characteristic rash. However, when this rash is not present, diagnosis is more difficult, and traditional tests may require potentially dangerous procedures for sick patients.

What did the researchers do? A newer type of test called next-generation sequencing (NGS) can help with this problem. This test can look for many different viruses and bacteria at the same time in the bloodstream of patients. In this study, researchers looked at the medical records of ten patients with low immune systems who tested positive for HHV8.

What did the researchers find? They found that this test could correctly decide if HHV8 was the cause of the illness in eight out of ten cases. In the other two cases, they found a different infection that was causing the person’s illness.

What do the findings mean? This study suggests that this test can be a helpful tool for diagnosing infections in immunocompromised patients. It can be faster and more accurate than routine tests.

## Background

Human herpesvirus-8 (HHV8) is a source of significant disease burden among patients with impaired cellular immunity. It is the causative agent for Kaposi sarcoma (KS), primary effusion lymphoma (PEL), multicentric Castleman’s disease (MCD), and Kaposi sarcoma inflammatory cytokine syndrome (KICS).^1^ HHV8-related illness tends to manifest in immunocompromised patients, and prompt identification of HHV8 disease can significantly impact prognostication and outcomes, particularly when antineoplastic treatment is indicated.^2^ Furthermore, patients can have multiple manifestations of HHV8-related illness at once, such as KS and MCD. Therefore, even if one illness has been found, diagnostic tools are required to identify concurrent illnesses that could require different treatment.^3^

When HHV8 is suspected to cause a presenting syndrome, clinicians can typically use serology or serum polymerase chain reaction (PCR) when obtaining initial laboratory studies. Serological assays are not currently recommended as a reliable test for identifying HHV8; they require initial suspicion of HHV8-related illness, which may not be apparent in patients with PEL, MCD, or KICS.^4,5^ Furthermore, the turnaround times (TATs) for serology and serum HHV8 are variable among performing laboratories, with advertised ranges from 8 h to 6 days.

Diagnosing HHV8-related illnesses often requires biopsies with immunohistochemistry (IHC) staining.^6^ These invasive diagnostics provide unique risks, including aspiration, bleeding, infection, and iatrogenic pneumothorax.^7,8^ Moreover, they are resource-intensive and take a long time to collect and process.

Next-generation sequencing of plasma microbial cell-free DNA (mcfDNA NGS) has recently entered the repertoire of diagnostic tools clinicians use to narrow the differential diagnosis of these challenging presentations. mcfDNA NGS is an open-ended and noninvasive assay that can identify thousands of potential pathogens.^9^ The mcfDNA NGS test utilizes an automatic liquid-handling platform to extract mcfDNA and prepare next-generation sequencing (NGS) libraries from a plasma sample. Pathogens are identified when NGS libraries are multiplexed, sequenced, and compared to known pathogenic DNA sequences.^10^

In this study, we reviewed the charts of 10 patients who had undergone mcfDNA NGS (Karius Test™). Our objective was to examine the role of this test as a diagnostic tool for illnesses caused by HHV8 in these patients’ clinical courses.

## Design and methods

This case series was conducted at Baylor St. Luke’s Medical Center, a quaternary care center in Houston, Texas. Patient charts were selected for review if they had positive mcfDNA NGS testing for HHV8 between 2017 and 2024.

At the institution where this study was conducted, mcfDNA NGS was a send-out modality by the brand name Karius Test to Karius (Redwood City, CA, USA). This test can identify over 1000 pathogens by their DNA in the blood. The reference range for HHV8 by the Karius is <10 DNA molecules per microliter (MPM). Pathological analysis and IHC stains were ordered based on clinician preference and conducted at the hospital laboratory. Serum HHV8 PCR was a send-out test for Quest Diagnostics (San Juan Capistrano, CA, USA) or Eurofins Viracor (Lenexa, KS, USA).

The medical records were analyzed by two researchers (R.B. and J.J.), who recorded information regarding clinical presentation, suspected diagnoses, laboratory studies (serum HHV8 PCR, serology, mcfFNA NGS results for HHV8 and any other organisms identified), procedures (thoracentesis and bronchoscopy), and pathology including IHC. Then, two researchers (M.A.M. and F.H.C.) cross-checked the initial chart review for accuracy.

Researchers calculated TATs for mcfDNA NGS testing and, when applicable, for HHV8 PCR and IHC. Clinical TAT describes the time between the clinician ordering the test and receiving the results. Laboratory TAT was defined as the time between the laboratory receiving the specimen and reporting the result.

Two researchers (R.B. and M.A.M.) identified changes in the final diagnosis by comparing the suspected diagnoses when mcfDNA NGS was ordered to the final diagnosis after the test resulted. Management changes were defined as any alterations in the antimicrobial regimen or specialty referral due to mcfDNA NGS result, per the medication administration record or the provider note.

## Results

All 10 patients were male, with a median age of 40.5 years (range: 27–68). They were all immunosuppressed: nine had human immunodeficiency virus (HIV; median CD4: 37, range: 4–139), and one had a history of liver transplantation. Before mcfDNA NGS testing, five patients with HIV were on antiretroviral therapy (Table 1).

**Table 1.** Clinical presentation and relevant objective findings.

Case number	1	2	3	4	5	6	7	8	9	10
Age (years), gender	49, Male	68, Male	47, Male	34, Male	39, Male	58, Male	33, Male	42, Male	28, Male	27, Male
Previous diagnosis of KS	No	Yes	No	Yes	Yes	No	No	No	No	Yes
HIV diagnoses	Yes	No	Yes	Yes	Yes	Yes	Yes	Yes	Yes	Yes
CD4 (cells/mm^3^)	67	NA	100	73	139	17	4	34	37	33
HIV viral load (copies/mL)	167	NA	160	70,000	137	698,000	13,000	150	1,050,000	Undetectable
Initial clinical symptoms	Fever, dyspnea, lymphadenopathy skin nodules	Fever, dyspnea	Nausea, vomiting, weight loss	Fever, dyspnea, hypoxia	Fever, severe pain, and lower right extremity edema	Fever, tachypnea, diarrhea, blurry vision, odynophagia	Fever, diffuse maculopapular rash, right eye endophthalmitis post right eye evisceration	Fever, weight loss, dyspnea, abdominal pain	Fever, cough	Worsening dyspnea after hospitalization PCP
Cutaneous lesions	Crusted nodules	None	None	Large fungating mass	Multiple nodular lesions	Purple nodule lesions	None	A rash is not typical of KS	None	Diffuse dark plaques
Bronchoscopy	A raised endobronchial lesion on the right main bronchus	Few small erythematous lesions near the main bronchus	NA	NA	NA	Normal appearing anatomy	NA	NA	NA	Normal appearing anatomy
Initial suspected diagnosis	Pneumonia (viral, atypical bacterial, mycobacterial, fungal, PCP)	Pulmonary KS	Disseminated MAC, or other opportunistic organisms	Multifactorial acute hypoxic respiratory failure (COVID-19)	Cutaneous KS	Cutaneous KS	Fever likely due to opportunistic organisms	Lymphoma or opportunistic organisms	Persistent fever of unknown etiology	Disseminated KS

HIV, human immunodeficiency virus; KS, Kaposi sarcoma; MAC, *Mycobacterium avium* complex; NA, not applicable/test not performed; PCP, *Pneumocystis jirovecii* pneumonia.

Clinicians suspected KS in five patients before mcfDNA NGS testing, including four with a previous diagnosis of cutaneous KS (three had the lesions still present on admission) and one with a new diagnosis (Table 1). The initial differentials were broad for the remaining five patients and did not include KS.

Compared to IHC and serum HHV8 PCR, mcfDNA NGS was the quickest positive laboratory result for HHV8 in all but two cases. The median clinical and laboratory TATs for mcfDNA NGS were 3 and 1 day, respectively. Six patients underwent IHC testing for HHV8, with a median clinical TAT of 8.5 and a median laboratory TAT of 7 days. Only two patient charts reviewed had send-out serum HHV8 PCR results with an average clinical TAT of 5.5 days and a laboratory TAT of 2 days.

After a clinical workup, eight patients had a final diagnosis related to HHV8 (Table 2). Five had a manifestation of KS: two (patients 5 and 6) had cutaneous KS, one (3) had pulmonary KS, and two (4 and 10) had both cutaneous and pulmonary KS. One patient (1) had either MCD or KS, and the final diagnosis was not ascertained due to a lack of follow-up. The two remaining patients with HHV8-related illnesses had KICS (patient 2) and PEL (patient 8).

**Table 2.** Results and comparison of microbiology testing for HHV-8 and impact on diagnoses.

Case number	1	2	3	4	5	6	7	8	9	10
Pathology	No malignant cells were identified on BAL or right main bronchus biopsy	Lymph node biopsy consistent with KS. Autopsy consistent with KS	FNA from right upper lung biopsy consistent with KS, positive HHV-8 IHC staining	Pleural fluid positive for reactive mesothelial cells and chronic inflammatory cells	Right leg excision skin biopsy consistent with KS	BAL positive for PCP negative for malignancy	Right eye biopsy with atypical lymphoid population, plasmacytic differentiation	Result after mcfDNA testing: Lymph node biopsy positive for PEL; positive stain for HHV-8 and EBV	Induced sputum and peritoneal fluid negative for malignancy and *Pneumocystis* organisms	Right lower lobe lung biopsy morphology consistent with KS, sputum negative for *Pneumocystis jirovecii*
Quantitative serum HHV-8 PCR (copies/mL)	NA	2,000,000	NA	6119	NA	NA	NA	NA	NA	NA
IHC	NA	NA	Conducted after mcfDNA testing: Positive for HHV-8, from right upper lobe biopsy	Negative for HHV-8, from pleural effusion	Positive for HHV-8, from skin biopsy	NA	Negative for HHV8, from the right eye	Positive for HHV-8 and EBV, from mediastinal lymph node	NA	Positive for HHV-8, from right lower lung biopsy
mcfDNA NGS—*HHV-8* mcfDNA NGS (MPM)	Positive (MPM not given)	134,105	595	164	18,911	183	27	44,270	826	1684
mcfDNA NGS—*Other organisms* mcfDNA NGS (MPM)	None found	None found	None found	None found	EBV (214)	CMV (316,000), PCP, (259,649) Adenovirus (1280)	Torque teno virus 6 (1546), *Helicobacter cinaedi* (141)	EBV (507)	CMV (117) *P. jirovecii* (75)	Not detectable
Change in final diagnoses after mcfDNA NGS result	Yes, possible pulmonary KS vs Castleman disease	Yes, KICS, in the setting of negative bronchoscopy which ruled out pulmonary KS, and clinical picture consistent with KICS	Yes, pulmonary KS	Yes, pulmonary KS, cutaneous KS was present but diagnosed prior to mcfDNA NGS	No, cutaneous KS was already diagnosed	No, cutaneous KS was already suspected	No, the diagnosis was indeterminate, no evidence of KS	Yes, HHV-8 and EBV-related PEL	Yes, extrapulmonary *P. jirovecii*, no evidence of KS	Yes, cutaneous KS was previously diagnosed but pulmonary KS was not
Did mcfDNA NGS result impact referrals made by clinicians?	Yes, referred to outpatient dermatology and gastroenterology	Yes, referred to oncology for chemotherapy	Yes, referred to oncology for chemotherapy	Yes, referred to oncology for chemotherapy	No	No	No	Yes, referred to oncology for chemotherapy	No	No, the patient received comfort care due to declining status
Change in antibiotic management	No	No	No	No	No	No	No	Yes, Valganciclovir for CMV stopped	Yes, TMP-SMX dose increased from prophylactic to treatment to cover PCP	No

BAL, bronchioloalveolar lavage; CMV, cytomegalovirus; EBV, Epstein-Barr virus; FNA, fine needle aspiration; HHV-8, human herpesvirus-8; IHC, immunohistochemistry; KICS, Kaposi sarcoma inflammatory cytokine syndrome; KS, Kaposi sarcoma; mcfDNA NGS, microbial cell-free DNA next-generation sequencing; MPM, DNA molecules per microliter; NA, not applicable/test not performed; PCP, pneumocystis jirovecci pneumonia; PCR, polymerase chain reaction; PEL, primary effusion lymphoma; TMP-SMX, trimethoprim/sulfamethoxazole.

The mcfDNA NGS changed the final diagnosis in seven patients and impacted referrals and/or antibiotic management in six (Table 2). In five of these patients, the diagnosis of HHV8-related illness resulted in an oncology or dermatology referral, and in one of them, anti-cytomegalovirus (CMV) therapy was discontinued. In the sixth patient, the mcfDNA NGS test identified an additional pathogen, *Pneumocystis jirovecii*, requiring treatment. The remaining patient had pulmonary KS diagnosed after the mcfDNA NGS result, but the family chose comfort care due to poor disease prognosis. There was no change in the diagnosis or management in three additional patients (two had cutaneous KS already suspected before the test, and one had no evidence of HHV8-related disease).

The median hospital stay was 19 days (6–31 days). Four patients died: three during the admission when mcfDNA NGS was performed and one 7 months after the index hospitalization when mcfDNA NGS was done.

## Discussion

This report found that mcfDNA NGS helped identify HHV8-related disease in severely immunocompromised patients and had a more pronounced impact on treatment plans for patients whose clinicians did not initially suspect HHV8 as the causative agent based on clinical presentation. Since HHV8-related illness can mimic many other diseases in the immunocompromised, this demonstrates that mcfDNA NGS is a promising tool for clinicians attempting to treat immunocompromised patients at risk for HHV8-related illness.

As seen in this study, some patients received mcfDNA NGS testing without PCR and/or IHC. mcfDNA NGS testing was warranted for these patients because they were severely immunocompromised with fever, meaning that the differential diagnosis was broad enough that it prevented clinicians from predicting disease-causing pathogens by clinical presentation alone.

In these nonspecific patient presentations, mcfDNA NGS offered an open-ended diagnostic tool to help direct further diagnostic workup as well as management and referrals.

This is the first study to identify mcfDNA NGS’s role in diagnosing HHV8-related illness. We found that the mcfDNA NGS diagnosis of HHV8 had clinical utility in 6 of 10 patients (60%) with HHV8-related illnesses. Although no other studies assessed HHV8 and mcfDNA NGS, several reports analyzing the role of mcfDNA NGS in immunocompromised people offer data for comparison.^11,12^ In a study of immunosuppressed pediatric patients due to hematopoietic stem cell transplant (HSCT) or hematologic or solid organ malignancy, the positive clinical impact (13%) was minimal compared to our study (60%).^13^ Differences in population characteristics could explain this difference: this case series included adults who were immunosuppressed primarily due to advanced AIDS.

The diagnostic performance of mcfDNA was evaluated by a systematic review of 6 studies involving 71 individuals with a history of HSCT. It found a high diagnostic utility (diagnostic odds ratio: 35.6) attributed to the high pre-test probability in the immunosuppressed population.^14^ Although we did not assess the accuracy of the test due to the small sample size and our patients were immunosuppressed due to HIV and solid-organ transplant instead of HSCT, we noted a high correspondence between positive mcfDNA NGS test results and clinical illness.

Since viral or bacterial shedding in immunosuppressed patients can result in positive molecular testing in the absence of a corresponding syndrome, diagnostic stewardship efforts are required to interpret results and avoid unnecessary antimicrobials, specialist referrals, and associated costs. In one retrospective study, 5.3% of pediatric patients underwent unnecessary diagnostic procedures or received antimicrobials based on positive results for pathogens that were not causing illness following mcfDNA NGS testing.^11^ Another study assessed the clinical utility in patients with pneumonia and found discordance between mcfDNA NGS testing and sputum culture in 3 out of 11 patients.^15^ In two cases, the mcfDNA NGS identified *Pseudomonas aeruginosa*; however, the patients improved without anti-pseudomonal therapy.

By contrast, this series only identified two patients who tested positive for HHV8 on mcfDNA NGS but did not have HHV8-related illness. Despite the positive results, these two patients did not undergo unnecessary procedures as they were attributed by the infectious disease provider as asymptomatic HHV8 viremia.^16^ Other authors have demonstrated complete agreement between pathogen identification via mcfDNA NGS and clinical adjudication in both immunocompromised and immunocompetent patients for certain pathogens and clinical scenarios.^17,18^ Given this variability in results, physicians must continue to exercise careful reasoning when interpreting mcfDNA NGS test results.

Our study has several limitations. The sensitivity of mcfDNA NGS for HHV8 was not ascertainable, given the lack of data for patients with negative mcfDNA NGS testing and positive testing by other modalities. Not all patients received PCR and IHC testing in addition to mcfDNA NGS, which makes it difficult to compare these modalities. The absence of a comparison arm prevented analysis of the test’s cost-effectiveness. Moreover, the study’s small sample size of patients testing positive for HHV8 by mcfDNA NGS and institutional variability in TATs limited the external validity of TAT estimates.

One strength of this study was the judicious care implemented when clinicians chose to order mcfDNA NGS testing. Given the patients’ immunocompromised status and evidence of active infection, all cases had a high pre-test probability. This may have contributed to the low rate of clinically irrelevant mcfDNA NGS results. Furthermore, the detailed chart review for each patient allowed for a comprehensive analysis that isolated the changes imparted by the mcfDNA NGS result on diagnoses and management within the clinical context of other testing methods.

## Conclusion

Identifying HHV8-related illnesses remains challenging in immunocompromised patients with nonspecific symptoms and vast differentials. mcfDNA NGS is an additional tool that can aid clinicians in identifying HHV8-related diseases and adjusting treatment plans, particularly in patients with nonspecific, extracutaneous symptoms. Further studies are needed to understand this test’s impact on patient outcomes.

## Declarations

**Ethics approval and consent to participate:** This study was ethically approved by Baylor College of Medicine institutional review board, approval number: H-45143. The Baylor College of Medicine institutional review board waived the informed consent to participate requirement, given the study was retrospective.

**Consent for publication:** The IRB board waived the consent requirement, given the study was retrospective.

**Availability of data and materials:** The data and materials accessed for this study contain identifiable patient information and are, therefore, only available to providers and for research purposes.
